# Intention to use contraceptives and its correlates among reproductive age women in selected high fertility sub-saharan Africa countries: a multilevel mixed effects analysis

**DOI:** 10.1186/s12889-023-15187-9

**Published:** 2023-02-06

**Authors:** Wubshet Debebe Negash, Habitu Birhan Eshetu, Desale Bihonegn Asmamaw

**Affiliations:** 1 Department of Health Systems and Policy, Institute of Public Health, College of Medicine and Health Sciences, PO.Box.196, University of Gondar, Gondar, Ethiopia; 2 Department of Health Promotion and Health Behavior, Institute of Public Health, College of Medicine and Health Sciences, PO.Box.196, University of Gondar, Gondar, Ethiopia; 3 Department of Reproductive Health, Institute of Public Health, College of Medicine and Health Sciences, University of Gondar, Gondar, Ethiopia

**Keywords:** Intention, Contraceptive, Reproductive age women, High fertility countries

## Abstract

**Background:**

Intention to use contraceptive methods has an overriding importance to better visualize the women’s future needs and more likely to translate it to actual behavior. It is therefore important to identify the motivating correlates such as education, women empowerment, as well as deterring factors like fear of side effects, infertility after contraceptive use, lack of knowledge regarding family planning methods among married women in countries with high fertility rates in sub-Saharan Africa. This helps to control family size, unintended pregnancies, and poor health outcomes for infants and mothers.

**Methods:**

A secondary data analysis was performed using the recent Demographic and Health Surveys. A total weighted sample of 178,875 reproductive age women was included in this study. A multilevel mixed-effect binary logistic regression model was fitted. The odds ratios along with the 95% confidence interval were generated to identify the correlates of the intention to use contraceptives. A p-value less than 0.05 was declared as statistical significance.

**Results:**

Overall, the intention to use contraception was 37.66% (95% CI, 37.44, 37.88). Whereas, the proportion of women who intend to use contraception was 59.20%, 53.30%, 42.32%, 37.88%, 37.63%, 35.25%, 31.32%, 20.64%, 20.30% in Burkina Faso, Burundi, Niger, Mali, DR. Congo, Nigeria, Angola, Gambia, and Chad respectively. Age; 15–24 (AOR = 3.72, 95% CI, 3.58, 3.86) and 25 − 24 years (AOR = 2.81, 95% CI, 2.74, 2.89), education of women; primary (AOR = 1.16, 95% CI, 1.13, 1.20), and secondary (AOR = 1.32, 95% CI, 1.27, 1.37), wealth index; middle (AOR = 1.15, 95% CI, 1.12, 1.18), rich (AOR = 1.28, 95% CI, 1.24, 1.32), number of living children 1–2 (AOR = 1.42, 95% CI, 1.37, 1.48), 3 or more (AOR = 1.77, 95% CI, 1.69, 1.85), age at cohabitation ≥ 18years (AOR = 1.37, 95% CI, 1.33, 1.40), heard family planning messages in the media (AOR = 1.47, 95% CI, 1.43, 1.50), history of ever terminated pregnancy (AOR = 1.13, 95% CI, 1.09, 1.17) and perceived distance to the health facility as not big problem (AOR = 1.16, 95% CI, 1.13, 1.19) were the correlates of intention to use contraceptives.

**Conclusion:**

The finding of the current study demonstrates that the intention of contraceptive use among reproductive age women in high fertility countries in SSA was relatively low as compared to previous studies. Thus, each national authority, especially in Chad and Gambia would be keen to know the level of contraceptive use intentions for their respective region, the drivers of contraceptive use intention and to map priorities for behavioral change. Any intervention strategy that promotes intention of contraceptive use should consider these factors for better success. Future researchers interested in the area should also address qualitative variables like socio-cultural factors, which might have an effect on intention of contraceptive use.

## Background

The intention of utilizing the contraceptive method is paramount for better understanding the future need of the woman and making her more likely to turn that intention into action [1–3]. It is widely believed that intentions predict behavior and that in many behavior change interventions, including those targeting contraceptive use, behavioral intentions are used to evaluate program effectiveness [4, 5]. However, in high fertility SSA countries, little evidences are available about modern contraceptive [6, 7].

In the aftermath of the 2012 London Summit on family planning, more than 40 countries worldwide recognized that life-saving contraception should be recognized as an essential human right for women [8]. Intention to use and use of family planning has been the best method to control family size and unintended pregnancies [9]. Despite the fact that intention to use family planning is known to improve maternal and child health, family and society at large [10, 11], in sub-Saharan Africa unintended pregnancies, high fertility, and abortion are still challenges for women of reproductive age [12, 13]. As the number of children rises to four or more, there will be an increase in the risk of maternal mortality [14].

To regulate high fertility, implementing a family planning strategy is very important [15]. By the year 2020, the London summit was aimed to mobilize service deliveries to the rights of an additional 120 million mothers in the world’s poorest countries to use contraceptives [16]. Achieving this objective enables us to prevent 100 million unintended pregnancies, 50 million abortions, 3 million infant deaths, and 21 thousand maternal death [17]. In countries of high fertility, knowing the intention is an important input for implementing a good strategy for family planning [18], and it is also used as an indicator of potential demand for family planning services [18–20].

In African countries, women’s intention to use contraception has been influenced by their partners’ fertility preference [21–23], parity [24], number of living children [25], misconceptions about contraceptives [26], and socio-demographic factors like marital status, residence, the number of children, age, religion [24, 27–30]. By considering these factors and adding other community level factors such as community level education, community level media exposure, distance to the nearest health facility assessing the intention to use contraception brings tangible evidence for intervention [31].

The effectiveness of contraceptives to manage the high fertility have been documented in specific sub-Saharan Africa (SSA) countries like in Ethiopia 44% [31], Ghana 31.7% [32], and Mozambique 44.7% [21]. Additionally, in SSA, studies on the intention to use contraceptives have been investigated by the same authors [33]; however, studies that combine in the context of specific high fertility countries [34] in SSA have not been carried out to understand the intention of women to use contraceptives and why low intention to use contraceptives. Thus, by using a multilevel mixed effect approach, we can better understand individual and community level correlates of intention to use contraception. This will in turn useful as input for program planners, resource allocators and governments to predict future behaviors and to be effective in their program implementation. Additionally, the study helps to develop an effective behavior change communication strategy and is an insight to demand and future use in the respective countries.

## Methods

### Study settings and data source

Community-based cross-sectional survey was conducted between January 2010 and December 2018 among reproductive age women in high fertility countries in sub-Sahara Africa (SSA). Niger, Democratic Republic of Congo, Mali, Chad, Angola, Burundi, Nigeria, Gambia, and Burkina Faso were included in this study. These countries were selected because they are the top ten countries with high fertility rates in SSA with fertility rates above 5.0, a value that is higher than the rate of 4.44 in Africa and 2.47 worldwide [34]. One country (Somalia) with no Demographic and Health Survey (DHS) data was excluded from this analysis.

The data for these countries were obtained from the official database of the DHS program, https://dhsprogram.com after authorization was granted via online request by explaining the purpose of our study. We used the woman record (IR file) data set and extracted the dependent and independent variables. DHS is a nationally representative household survey that was conducted across low and middle-income countries every five years [35]. It has been an essential data source on issues of reproductive health in low and middle-income countries as it gathers data on several reproductive health issues [35].

Study participants were selected using a two-stage stratified sampling technique. Enumeration Areas (EAs) were randomly selected in the first stage while households were selected in the second stage. A total weighted sample of 178,875 married reproductive age women were included in the study. Women with infecundity, current contraceptive users and currently pregnant were excluded [36]. Details about the selected countries, the year of the survey, and the sample size are shown in Table 1.

**Table 1:** Sample size determinations of intention to use contraceptives high fertility sub Saharan Africa countries: based on 2010–2018 DHS

Countries	Survey year	Unweighted sample(n)	Weighted sample(n)	Percentage (%)
Angola	2015/16	7228	6869	3.84
Burkina Faso	2010	22,344	22,738	12.71
Burundi	2016/17	13,742	13,979	7.81
DR Congo	2014/15	20,420	19,093	10.67
Chad	2013/14	24,902	24,238	13.55
Gambia	2013	12,558	12,271	6.86
Mali	2018	14,042	14,180	7.93
Nigeria	2018	48,702	48,493	27.11
Niger	2012	16,036	17,014	9.51

### Variables of the study

#### Dependent variable

The dependent variable for this study was intention to use contraceptives among reproductive-age women. It was categorized as “yes” for those women who had intention to use latter and coded it as “1” whereas, those women who were unsure about use or not intended to use were grouped under “no” and coded as “0” [33, 37, 38].

#### Independent variables

The analysis incorporated several individual and community level independent variables based on available evidence on the intention of contraception use among reproductive-age women (Table 2).

**Table 2:** List of variables for the assessment of quality of ANC among pregnant women in Ethiopia

Variables	Description
Individual level variables	
Age of the women in year	15–24, 25–34, and 35–49
Women educational level	No formal education, Primary education, and Secondary and higher education
Women occupation	Working, not working
Husband educational level	No formal education, Primary education, and Secondary education and higher
Wealth index	In the DHS data, wealth index is categorized into quintile as poorest, poorer, middle, richer, richest using principal component analysis. A high degree of variability in observation from the original DHS classification led to the re-categorization of wealth index scores into three categories: poor, middle, and rich, which was categorized by merging the poorest with the poorer and the richest with the richer for easier interpretation [36].
Heard family planning messages in the media Ideal number of children	Coded “yes” if the women heard/read family planning messages either in newspaper, radio, or television for at least less than once a week, and “no” for otherwise. < 4, ≥4
age at first cohabitation	< 18, ≥ 18
Number of living children	None, 1–2, ≥3
Ever had terminated pregnancy **Community level variables**	Yes, No
Place of residence	Rural, Urban
Distance to the nearest health facility	Participants were asked if distance to the health facility is a problem to them or not. Their response was categorized as “Big problem”, “Not big problem” [36].
Survey year	2010–2013, 2014–2015, 2016–2018
community level education and community level media exposure	The aggregate community level independent variables (community-level education and community level media exposure) were constructed by aggregating individual-level characteristics at the community (cluster) level. These were categorized as high or low based on the distribution of the proportion values computed for each community after checking the distribution by using the histogram. The aggregate variables were not normally distributed and the median value (50%) was used as a cut-off point for the categorization of each community level variables. Finally, categorized as low if the proportion from a given community is < 50% and high if the proportion is ≥ 50%. All the independent variables were identified from reviewing different literatures [33, 37–40].

### Data analysis

Stata version 14 statistical software was used for data analysis. The data were weighted throughout the analysis to ensure the representativeness of the DHS sample and get reliable estimates and standard errors before data analysis. To maintain the representativeness of the sample data, we used weighted values before analyzing the DHS dataset, since the overall probability of selecting a household is not constant. We chose the individual weight for women (v005), which is the household weight (hv005) multiplied by the inverse of the individual response rate for women in the stratum. To calculate individual sample weights, divide (v005) by 1,000,000, then use it to estimate the number of cases [41]. Out of 179,974 total eligible households, 178,818 had complete data on intention to use modern contraceptives with a response rate of 99.4%. Overall, a total weighted sample of 178,876 married reproductive age women from all nine countries were included in this study. Four models were fitted in this study: the null model, which had no explanatory variables, model I, which had individual-level factors, model II, which had community-level factors, and model III, which had both individual and community-level components. Since the models were nested, the Intra-class Correlation Coefficient (ICC) and Median Odds Ratio (MOR) were used for model comparison. On the other hand, deviance (-2LLR) values were used for model goodness of fit. Model III was chosen as the best-fitted model since it had the lowest deviance. Variables having a p-value less than 0.2 in bivariable were used for multivariable analysis. Finally, in the multivariable analysis, adjusted odds ratios with 95% confidence intervals and a p-value of less than 0.05 were used to identify correlates of intention to use contraceptives.

## Results

## Socio-demographic and economic characteristics

A total weighted sample of 178, 875 married reproductive age women (15–49) were included in the survey. The median age of the women was 30 years (IQR: 24, 37). Nearly three-fourths (73.60%) of the women were from rural areas. Most of the women (61.35%) had perceived the distance to the health facility as not a big problem (Table 3).

**Table 3:** socio-demographic and economic characteristics of respondents in high fertility countries in sub-Saharan Africa (n = 178,875): based on 2010–2018 DHS

Variables	Categories	Frequency	Percentage
Age in years	15–24	47,416	26.51
	25–34	70,352	39.33
	35–49	61,107	34.16
Residence	Urban	47,222	26.40
	Rural	131,653	73.60
Educational status of respondents	No formal education	107,605	60.16
	Primary education	36,466	20.39
	Secondary & Higher education	34,804	19.46
Husband education			
	No formal	96,721	54.12
	Primary	3646	17.15
	Secondary and higher	51,343	28.73
Occupation of respondents(164,969)	Not working	53,852	32.64
	Working	111,117	67.36
Wealth index	Poor	78,024	43.62
	Middle	37,291	20.85
	Rich	63,561	35.53
Mass media exposure	Yes	103,044	57.80
	No	75,248	42.20
Distance to the health facility	Big problem	62,632	38.47
	Not big problem	100,197	61.35

### Obstetrics-related characteristics of mothers

The majority (83.92%) of the women had four or more ideal number of children. More than half (58.72%) of the women had cohabitation with their partners before the age of 18 years (Table 4).

**Table 4:** Obstetrics-related characteristics of mothers in selected high fertility sub-Saharan Africa countries (n = 178,875): based on 2010–2018 DHS

Variables	Categories	Frequency	Percentage
Ideal number of children	< 4	28,764	16.08
	≥ 4	150,111	83.92
Age at first cohabitation	< 18	105,031	58.72
	≥ 18	73,844	41.28
Number of living children	None	15,880	8.88
	1–2	55,138	30.83
	≥ 3	107,857	60.30
Ever had terminated pregnancy	Yes	27,224	15.23
	No	151,588	84.34

### Intension to use contraceptive

**Fig. 1:**
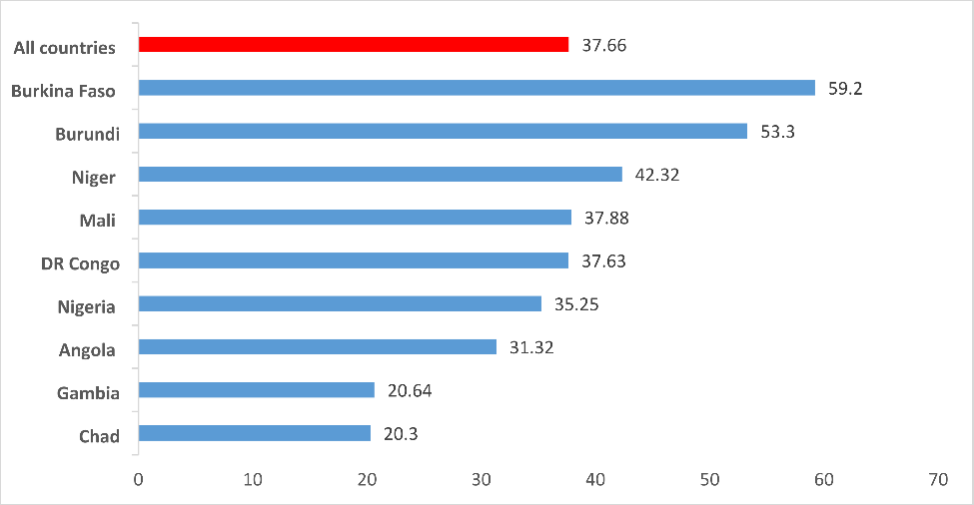
Intention to use contraception in high fertility sub-Saharan Africa countries: based on 2010–2018 DHS

Overall, 37.66% (95% CI, 37.44, 37.88) of the women had intention to use contraceptives. Women from Chad has the lowest (20.3%) intention to use contraceptive (Fig. 1).

### Fixed effects (a measure of association) results

The third model is the complete model that shows the correlates of individual and community-level factors of intention to use contraceptives among reproductive age women. Age of the women, educational status of women, wealth index, number of living children, age at cohabitation, heard family planning messages in the media, ever terminated pregnancy, and distance to the health facility were correlates of intention to use contraceptives among reproductive age mothers.

Accordingly, women whose age was 15–24 years (AOR = 3.72, 95% CI, 3.58, 3.86) and 25-24years (AOR = 2.81, 95% CI, 2.74, 2.89) had 3.72 and 2.81 times higher odds of intention to use contraceptives than those aged 35 and above, respectively. Women who had learned primary (AOR = 1.16, 95% CI, 1.13, 1.20) and secondary/higher education (AOR = 1.32, 95% CI, 1.27, 1.37) had 1.16 and 1.32 times higher likelihood of intention to use contraceptives relative to non-educated women. Regarding the wealth index; those women in the middle (AOR = 1.15, 95% CI, 1.12, 1.18), and rich (AOR = 1.28, 95% CI, 1.24, 1.32) wealth categories had 1.15 and 1.28 times higher likelihood of intention to use contraceptives than women from the poor wealth quantile, respectively. Women who had one to two children had 1.42 (AOR = 1.42, 95% CI, 1.37, 1.48) times and 3 and above 1. 77 (AOR = 1.77, 95% CI, 1.69, 1.85) times high likelihood to use contraceptives than those women with zero births.

The likelihood of intention to use contraceptives had higher by 1.37 among women who had cohabitation with their partner after the age of 18 years and above than women who had married below 18 years old (AOR = 1.37, 95% CI, 1.33, 1.40). The likelihood of intention to use contraceptive has higher by 47% (AOR = 1.47, 95% CI, 1.43, 1.50) among women who had media exposure than their counterparts. Reproductive age women who had a history of ever terminated pregnancy had 1.13 (AOR = 1.13, 95% CI, 1.09, 1.17) times higher likelihood of intention to use contraceptive than their counterparts.

With regard to the community level factors, the odds of intention to use contraceptives among reproductive age women who perceive distance to the health facility as not a big problem had 1.16 (AOR = 1.16, 95% CI, 1.13, 1.19) times higher compared to their counterparts. The odds of intention to use contraceptives among women had lower by 77% (AOR = 0.23, 95% CI, 0.22, 0.24) and 55% (AOR = 0.45, 95% CI, 0.44, 0.46) in the year 2013–2015 and 2016–2018, respectively in comparison with women in the 2010–2013 period (Table 5).

### Random effects (a measure of variation) results

In the baseline model without any correlate variable, 9.86% of the variance in the intention to use contraceptives could be explained by between-cluster variation of the characteristics (ICC = 0.098). The between-cluster variation was decreased to 8.75% in the final model (model 3), which had both the individual and community level factors. According to this, the variation in the likelihood of intention to use contraceptives could be attributed to differences in clusters.

Concerning the goodness of the model, model 3, which incorporated both the individual and the community level correlates, was chosen to predict intention to use contraceptives among reproductive age women. This model was selected because it has the lowest (201052.7) deviance as compared with the rest of the models (Table 5).

**Table 5:** Multilevel logistic regression models for individual and community correlates of intention to use contraceptives: based on 2010–2018 DHS

Variables	Intention to use contraceptive			Model 1 AOR (95% CI)		Model 2 AOR (95% CI)		Model 3 AOR (95% CI)		
	Yes n (%)		No n (%)							
**Individual level factors**										
**Age in years**										
15–24	21,016(44.32)		26,400(55.68)	3.50(3.38,3.63)				3.72(3.58,3.86)*		
25–34	31,184(44.33)		39,168(55.67)	2.70(2.63,2.77)				2.81(2.74,2.89)*		
35–49	15,163(24.81)		45,944(75.19)	1				1		
**Educational status of the women**										
No formal education	35,868(33.33)		71.737(66.67)	1				1		
Primary education	15,033(41.22)		21,433(58.78)	1.20(1.16,1.23)				1.16(1.13,1.20)*		
Secondary and higher	16,463(47.30)		18,341(52.70)	1.34(1.29,1.39)				1.32(1.27,1.37)*		
**Educational status of husband**										
No formal education	32,431(33.53)		64,290(66.47)	1				1		
Primary education	12,817(41.82)		17,829(58.18)	1.25(1.21,1.29)				1.02(0.97,1.05)		
Secondary and higher	22,066(42.98)		29,277(57.02)	1.05(1.02,1.08)				1.01(0.98,1.04)		
**Wealth index**										
Poor	26,615(34.11)		51,409(65.89)	1				1		
Middle	14,491(38.86)		22,800(61.14)	1.09(1.06,1.13)				1.15(1.12,1.18)*		
Rich	26,258(41.31)		37,302(56.69)	1.08(1.05,1.11)				1.28(1.24,1.32)*		
**Ideal number of children**										
< 4	10,306(35.83)		18,458(64.17)	1				1		
≥ 4	57,058(38.01)		93,053(61.99)	1.10(1.07,1.13)				1.03(0.98,1.06)		
**Number of living children**										
None	5854(36.87)		10,025(63.13)	1				1		
1–2	23,777(43.12)		31,362(56.88)	1.41(1.35,1.47)				1.42(1.37,1.48)*		
≥ 3	37,732(34.98)		70,124(65.02)	1.74(1.67,1.82)				1.77(1.69,1.85)*		
**Age at cohabitations (Years)**										
< 18	36,629(34.87)		68,402(65.13)	1				1		
≥ 18	3734(41.62)		43,110(58.38)	1.39(1.36,1.42)				1.37(1.33,1.40)*		
**Media exposure**										
No	23,176(30.80)		52,072(69.20)	1				1		
Yes	44,042(42.74)		59,002(57.26)	1.63(1.59,1.66)				1.47(1.43,1.50)*		
**Ever terminated pregnancy**										
No	56,675(37.39)		94,913(62.61)	1				1		
Yes	10,677(39.22)		16,547(60.78)	1.17(1.13,1.20)				1.13(1.09,1.17)		
**Survey year**										
2010–2013	20,661(51.98)		19,091(48.02)			1		1		
2014–2015	15,736(26.51)		43,628(73.49)			0.25(0.24, 0.26)		0.23(0.22, 0.24)*		
2016–2018	30,967(38.82)		48,793(61.18)			0.48(0.77, 0.88)		0.45(0.44, 0.46)*		
**Community level media exposure**										
Low	31,765(35.50)		57,712(64.50)			1		1		
High	3599(39.82)		53,799(60.18)			1.06(0.97,1.14)		0.92(0.85,1.02)		
**Community level education**										
Low	30,279(35.09)		56,017(64.91)			1		1		
High	37,085(40.06)		55,494(59.94)			1.17(1.08,1.26)		1.03(0.94,1.12)		
**Distance to the health facility**										
Big problem	22,597(36.08)		40,035(63.92)			1		1		
Not .Big problem	41,525(41.44)		58,672(58.56)			1.22(1.19,1.25)		1.16(1.13,1.19)*		
**Place of residence**										
Urban	19,158(40.57)		28,064(59.43)			1		1		
Rural	48,206(36.52)		83,447(63.38)			0.98(0.95,1.02)		1.04(0.95,1.14)		
**Random effect results**			**Null model**	**Model 1**		**Model 2**		**Model 3**		
ICC		9.86			9.32		9.13		8.75	
Variance (%)		36.0			33.81		33.1		31.55	
MOR		1.77			1.74		1.73		1.71	
PCV		Ref			6.08		8.06		12.36	
**Model fitness**										
Deviance(-2LL)		231053.42			217969.38		212388.66		201052.7	

* Statistically significant at p-value < 0.05, AOR: Adjusted Odds Ratio, COR: Crude Odds Ratio, Null model: without any determinant variables, Model 1: adjusted for individual-level variables, Model 2: adjusted for community-level variables, Model 3: adjusted for both individual and community-level variables, ICC: Intra class correlation, MOR: Median odds ratio, PCV: Proportional change in variance

## Discussion

This study investigated the intention to use contraception and its correlates among reproductive age women in high fertility countries in SSA. In the current study, nearly four in ten women, had intention to use contraception in high fertility countries. Of the high fertility countries, Burkina Faso (59.20%) had the highest prevalence of intention to use contraceptives. Chad (20.30%) and Gambia (20.64%) had the lowest prevalence of intention to use contraceptives. The higher intention to use contraceptive might be because In Burkina Faso, the Health System created a favorable environment through programs such as Engender Health for the provision of sexual and reproductive health to expand access to high-quality Family planning. Moreover, the Ministry of Health of Burkina Faso had supported by the civil society organizations and research institutions, international nongovernmental organizations, and United Nations (UN) agencies, for the development of a robust, responsive, and resilient health system [42]. Over 85% of women in Burkina Faso have access to their contraceptive method through the public sector, regardless of wealth quintile [43].

The result of the study showed that age of the women, level of education of the women, wealth index, media exposure, age at cohabitation, number of living children, ever had a terminated pregnancy, and distance to the health facility were the correlates of the intention to use contraceptives among reproductive age women in high fertility countries in SSA.

The finding of this study reveals that the overall prevalence of intention to use contraception is higher than individual studies conducted in Ghana 31.70% [32] but lower than studies conducted in Ethiopia 44.11% [31, 44], Mozambique 44.70% [21], and Pakistan 42.00% [45]. The low prevalence of intention to use contraceptives in this study compared to previous studies might be due to the difference in the study setting, sample size, and socio-demographic characteristics of the women. For instance, this study focuses on the top ten high fertility countries in SSA, whereas, the previous studies were done at the country level/individual countries. In terms of socio-demographic variations among respondents, previous study done in Mozambique reported that 38.54% of reproductive age women have no formal education, which was lower than that of the current study (60.16%). Previous literatures found that level of education has a positive relationship with intention on contraceptive use [21]. Hence, expansion of women’s education is recommended to increase awareness and intention on contraceptive use among reproductive age women.

In this study, respondents who were 15–24 and 25–34 years old were more likely to have an intention of contraception use than older age groups. It is consistent with the studies conducted in Ethiopia [31], Jordan [46], and Malawi [47]. It indicates that the proportion of women who have the intention of contraceptive use increases until it reaches its peak in the 30–34 age group. The possible explanation might be that women in the 15–24 and 25–34 age groups are the time at which most women engage in different activities to fulfill their needs. As a result, they want to postpone their birth. This implies that they might have more intention to use contraceptives [48, 49]. Moreover, there is a low risk of conception as women’s age increases [46].

The odds of intention to use contraceptives was higher among those women who had formal education have higher odds of intention to use contraceptives compared to those women who had no formal education. This finding is similar with those of studies conducted in Ethiopia [25, 31, 50] and Uganda [51]. Women with formal education are more likely to be exposed to contraceptives through the media, which increases their awareness of contraceptives and helps them understand how contraception can help with fertility reduction, unintended pregnancy, unsafe abortions, and other health concerns of mothers and children [52, 53]. Furthermore, educated women are more likely to choose their own contraception [53, 54]. Accordingly, educating women will help to improve contraception intention in these high-fertility countries in sub-Saharan Africa.

The likelihood of intention on contraceptive use among women from households with middle and rich wealth quintile was higher than those from households with poor wealth quintile. This finding is supported by a study done in Mozambique [55]. The reason might be women from rich households can be able to deal with the cost barrier associated with access to contraceptive use as compared to those from poor households since they can able to overcome both the direct and indirect costs associated with contraceptive uptake [56]. Another possible reason could be due to an income increase, exposure to different information, and financial accessibility of services will be improved [57].

In this study, women who had family planning messages in the media had higher odds of intention to use contraceptives compared to their counterparts. The same is true with a study conducted in Jordan [46]. The reason for this might be that women who had family planning messages in the media might have a better understanding of contraception, which can bring a positive change in their attitude towards contraception and have a substantial positive effect on contraceptive use and intended future use of contraception [58, 59]. The study suggested that family planning messages in the media would reduce the barriers to access and use of health care services, including future intentions of contraception.

Women who have more children have higher odds of intention to use contraceptives than their counterparts. This finding was in agreement with studies done in Ethiopia [31], and Jordan [46]. The possible reason might be that, as the number of children increases, women intend to use contraceptives, as their desired number of children will be met [31, 60]. Women with no experience of terminated pregnancy were found to be more likely to have the intention to use contraceptives. The reason could be that women who have no history of terminating pregnancies were more likely to meet their ideal number of children. The more living children they have, the more likely they intend to use contraception [46, 50].

Another important factor that significantly influenced the intention of contraceptive use in this study was age at cohabitation. Women whose age at cohabitation was 18 years or older were 1.37 times more likely to have an intention of contraceptive use compared with their counterparts. The finding of this study is in agreement with a study conducted in Ethiopia [31]. The possible reasons might be that women who had married at age 18 and above were more educated, from rich households, and had a good awareness of the health benefits of the contraceptive in reducing fertility, unwanted pregnancy, and other maternal and child problems [61, 62].

The odds of intention to use contraceptives among women had lower in the recent year than in the previous period. This might be in the recent year, those who have not currently used a method to become pregnant, those who are trying to become pregnant, those who have recently given birth, and those who are amenorrheic might be a higher proportion than in the previous year’s [63]. This implies the intention to use contraceptives has contributed to a decline in high fertility and a more rational approach to designing family planning interventions.

Furthermore, women who perceive the distance to the health facilities as not a big problem were more likely to intend to use contraceptives as compared to their counterparts. The possible explanation could be that women who perceives the distance to the health facilities as not a big problem have good awareness of contraception since they are more likely to receive counseling on family planning and receive the recommended maternal health care services [64]. In addition, previous reports found that distance to healthcare facilities is an important deterrent for women seeking healthcare services [65, 66].

The study’s main strength was that it used nationally representative survey data. In addition, the DHS uses validated instruments in its appraisals of datasets along with its large sample size and well-designed procedures, such as training field enumerators and employing well-tested methods for data collection. However, since DHS data did not include qualitative data, we are unable to address the association of qualitative variables such as socio-cultural factors to the intention of contraception use. The measure of contraceptive use intention (an affirmative response to whether women intend to use contraceptives) less meaningful with no consideration for time. It will be better for future researchers to consider the time when they are intending to use contraceptives.

## Conclusion

The finding of the current study demonstrates that the proportion of women who intend to use contraceptive is low in high fertility countries in SSA. Therefore, each national authority would be keen to know the level of contraceptive use intentions for their respective region, the drivers of contraceptive use intention and to map priorities for behavioral change. Age of the women, level of education of the women, wealth index, hearing family planning messages in the media, age at cohabitation, number of living children, ever had a terminated pregnancy, and distance to the health facility were the correlates of the intention of contraceptive use among reproductive age women. Any interventional strategy that promotes the intention of contraceptive use should consider these factors for its better success. Future researchers interested in the area better to address qualitative variables like socio-cultural factors and timing when they will use contraceptives which might have a tremendous effect on the intention of contraceptive use.

## Data Availability

Data for this study were sourced from Demographic and Health surveys (DHS), which is freely available online at (https://dhsprogram.com) or can be accessed online through an application to MEASURE DHS. Analysis syntax and outputs generated for the study can be made available upon request to the corresponding author.

## List of Abbreviations

AOR: Adjusted Odds Ratio
CI: Confidence interval
DHS: Demographic and Health Survey
ICC: Intra-class Correlation Coefficient
MOR: Median Odds Ratio
PCV: Proportional Change in Variance
SD: Standard Deviation
SSA: Sub-Saharan Africa
WHO: World Health Organization.
